# Retraction: Biomechanical Characteristics of Hand Coordination in Grasping Activities of Daily Living

**DOI:** 10.1371/journal.pone.0151685

**Published:** 2016-03-04

**Authors:** The PLOS ONE Staff

Following publication, readers raised concerns about language in the article that makes references to a 'Creator', and about the overall rationale and findings of the study.

Upon receiving these concerns, the *PLOS ONE* editors have carried out an evaluation of the manuscript and the pre-publication process, and they sought further advice on the work from experts in the editorial board. This evaluation confirmed concerns with the scientific rationale, presentation and language, which were not adequately addressed during peer review.

Consequently, the *PLOS ONE* editors consider that the work cannot be relied upon and retract this publication.

The editors apologize to readers for the inappropriate language in the article and the errors during the evaluation process.
